# A combined neural ODE-Bayesian optimization approach to resolve dynamics and estimate parameters for a modified SIR model with immune memory

**DOI:** 10.1016/j.heliyon.2024.e38276

**Published:** 2024-09-24

**Authors:** Donglin Liu, Alexandros Sopasakis

**Affiliations:** Department of Mathematics, Lund University, 22362 Lund, Skåne, Sweden

**Keywords:** SIR model, Neural ordinary differential equations, Forecasting, Parameter estimation, Time-delay loss of immunity

## Abstract

We propose a novel hybrid approach that integrates Neural Ordinary Differential Equations (NODEs) with Bayesian optimization to address the dynamics and parameter estimation of a modified time-delay-type Susceptible-Infected-Removed (SIR) model incorporating immune memory. This approach leverages a neural network to produce continuous multi-wave infection profiles by learning from both data and the model. The time-delay component of the SIR model, expressed through a convolutional integral, results in an integro-differential equation. To resolve these dynamics, we extend the NODE framework, employing a Runge-Kutta solver, to handle the challenging convolution integral, enabling us to fit the data and learn the parameters and dynamics of the model. Additionally, through Bayesian optimization, we enhance prediction accuracy while focusing on long-term dynamics. Our model, applied to COVID-19 data from Mexico, South Africa, and South Korea, effectively learns critical time-dependent parameters and provides accurate short- and long-term predictions. This combined methodology allows for early prediction of infection peaks, offering significant lead time for public health responses.

## 1 Introduction

The COVID-19 pandemic, which emerged in late 2019, unleashed devastating consequences worldwide, causing significant loss of life and profoundly affecting global economies and societal structures. In particular, the virus has shown its ability to reinfect populations and undergo evolutionary changes, leading to the occurrence of multiple infection waves [1]. Accurate and long-term forecasting of these waves can play a crucial role in helping healthcare professionals and first response organizations proactively prepare for and respond effectively to outbreaks. Such forecasting capabilities can help with the optimal allocation of resources, ultimately reducing the detrimental effects on public health, mortality rates, and the economy [2].

Despite persistent challenges in accurately predicting contagion dynamics [3], the widely used SIR model [4], [5] has been extensively used to investigate COVID-19 outbreaks using data from various countries around the world [3], [6], [7], [8], [9], [10]. However, there are inherent uncertainties associated with accurately capturing epidemiological dynamics. These uncertainties stem from the novelty of the virus, including unknown transmission features, mutations, and pathogen behavior, among other factors. Similarly, estimating model parameters from infectious data can be challenging. Data can be inaccurate, especially during a pandemic, due to inherent reporting delays. Furthermore, due to a lack of testing or even lack of testing facilities altogether, information about the number of infectious individuals can be incomplete. Consequently, these factors can hinder the effectiveness of models in capturing the intricacies of infection dynamics [11], which requires the exploration of alternative modeling strategies to address these challenges.

To address these challenges, we propose a modified SIR model that includes a time-varying reproduction number and time-delayed infection effects through a memory mechanism. Our approach integrates a Neural Ordinary Differential Equation (NODE) framework with Bayesian optimization to estimate critical time-dependent parameters accurately. This combined methodology leverages the strengths of both neural networks and Bayesian optimization to improve the descriptive and predictive capabilities of the model.

Our study aims to address some of the challenges associated with explaining the dynamics of a prolonged multiwave epidemic. In particular, our approach involves identifying a proper time-varying reproduction number, as well as including time-delayed infection effects through a memory mechanism in a modified SIR model for the COVID-19 pandemic. Specifically, we propose a modified integro-differential equation model incorporating time-delayed memory of infections similar to [12]. In contrast to [12], however, we resolve the model dynamics through a novel version of the NODE approach, which allows us to estimate some of the critical time-varying model parameters [13]. For example, a time-varying reproduction number has been shown to be essential for long-term forecasting involving multiple waves of the disease [8], [14], [15], [16]. To avoid the well-known ill-posed nature of the classic SIR model when time-dependent parameters are introduced [17], [18], we propose a Bayesian optimization technique combined with a deep learning optimizer which allows us to improve the optimization process.

The SIR model has been used in various settings to better understand the dynamics of COVID-19. In [17] the authors fit and compare nine different types of SIR models with COVID-19 data from the USA and Italy to infer unknown model parameters as well as unobserved model dynamics through machine learning approaches. In one of these models, a time-delay memory mechanism is also incorporated. However, running the model forward in time leads to erroneous dynamics since the solution is not unique [17].

The success of any such infection model depends on the accuracy of some of its critically important parameters. There are several ideas and discussions in [19], [20] on novel mathematical models and methods for expressing uncertainty in predictions. In [21], an SIR model with a sliding window approach (7 days) was used to allow one of the critical model parameters, the basic reproduction number $R_{0}$, to change.

Alternative formulations for $R_{0}$ exist in the literature, particularly in models that account for non-Markovian dynamics. For example, models that incorporate delay differential equations or integral equations can describe the spread of an epidemic more accurately by considering the time distribution of infectious periods or the effect of latency in the transmission dynamics. Streftaris and Gibson (2012) provide a comprehensive overview of such models and highlight their advantages in capturing the temporal aspects of disease spread that Markovian models may overlook. They show that non-Markovian models, through the inclusion of time delays and integral forms, “can better capture the temporal aspects of disease spread” during the epidemic process, particularly in scenarios where the assumption of exponentially distributed infectious periods is unrealistic [22].

Incorporating memory effects or time delays can lead to more accurate predictions, especially in cases where the infectious period or the incubation period of the disease significantly influences the transmission dynamics. For example, models with a gamma-distributed infectious period, as discussed by Lloyd (2001), offer a more flexible framework compared to the exponential distribution assumed in Markovian models [23]. Such models allow for a more realistic representation of the variance in the infectious period among individuals.

Furthermore, age-structured models or models with varying transmission rates over time (e.g., due to changing contact patterns or intervention measures) provide additional layers of complexity that can improve the precision of $R_{0}$ estimates. The work by Keeling and Rohani (2008) explores these aspects and provides a detailed examination of the impact of different assumptions on the calculation of $R_{0}$ [24].

In [15], an extended Kalman filter is used together with a SIRD model, which also tracks the number of dead individuals, to predict the dynamics of the spreading of COVID-19. However, the estimated model parameters are shown to vary wildly during some stages of the disease. As a result, the new daily reported number of infections as well as the number of susceptible and removed individuals reported in that study also display unrealistic profiles. A modified SEIR compartmental model is implemented in [16] which also employs Bayesian inference to estimate the model parameters. Similarly, in [25], the Bayesian Markov Chain Monte Carlo (MCMC) algorithm is utilized to estimate the parameters of an SEIR model for COVID-19. In the machine learning approaches proposed in [9], [26] extra derivatives are included in the loss function of a neural network to help resolve evolution and important parameters for their respective SIR models.

Allowing time-dependent model parameters is one way to improve the descriptive capability of epidemiological models. Another effective approach is to incorporate memory into model dynamics, which has been shown to better capture multiple infection waves and crucially describe the spread of infection [12], [27], [28]. In particular, [12] demonstrates how modeling immune memory decay—the gradual loss of immunity gained through infection—can simulate the multiwave dynamics observed in epidemics. This model accounts for the fact that, over time, removed individuals lose their immunity and transition back to a susceptible state, leading to subsequent waves of infection. Furthermore, [27] highlights the role of waning immunity gained by vaccination, emphasizing that the reduction in immunity levels over time can generate successive waves of infection and significantly impact epidemic trajectories. Additionally, [28] provides a detailed analysis of a time-delay system that focuses on the infectious duration and its impact on epidemic dynamics, incorporating the constant inflow rate of susceptible individuals as a parameter. In contrast, [12] models the inflow of susceptible individuals by considering the role of immune memory decay, showing how individuals transition from a removed to a susceptible state and thereby generate multiple waves of infection.

To account for long-range memory decay effects [29] employs fractional derivatives. Similarly, to account for long-term memory, [30] considers an SIRS-type (Susceptible-Infectious-Removed-Susceptible) model with fixed parameters to model COVID-19 in Malaysia. However, this model is not used to forecast, but rather to understand the rebound effect in transmission dynamics. In [31] infection delay effects due to quarantine are modeled through an additional policy factor (see Eq. (11) in that article) in the SIR model. This policy factor was trained on real data and fitted through a neural network. Another approach using integrodifferential equations [32] considers the delay effects of contagion and the decrease in susceptible populations due to recovery. Similarly, the authors in [12] propose a modified SIR model with an added integral term that emulates memory for infection dynamics. However, computation of the model parameters is not included in that work. To our knowledge, there are no studies that estimate these parameters based on the neural ordinary differential equations (NODEs) approach [13].

The main contributions of our work can be summarized as follows:

• Solving the inverse problem of estimating time-dependent model parameters in a time-delay SIR equation;
• Employing Bayesian optimization methods to improve the accuracy of our model for long-term dynamics;
• Providing short-term (days) and long-term (weeks or months) inference capabilities for analyzing the multi-wave COVID-19 infection dynamics.

There are several challenges in working with real population data during a pandemic. The exact number of infected individuals in the population will never be known. Furthermore, delays and errors in collecting and reporting information about the estimated number of infected individuals can negatively impact models [17], [33]. As a consequence of these challenges, we first test our model on synthetic data in order to verify that we can reproduce all the crucial model parameters since we know their true values. Then, to better understand how the model performs with data, which may include reporting delays or other errors, we also evaluate it using real data from Mexico, South Africa, and South Korea.

The remainder of this paper is organized as follows. In Section 2, we introduced the modified SIR model and how we estimate its parameters. A brief description of the datasets is given in Section 3. The computational results of the proposed method on these datasets and discussions are presented in Section 4. Our conclusions are provided in Section 5.

## 2 Methods

The SIR model is a fundamental tool for studying the dynamics of infectious diseases [34]. It provides a simple framework for understanding how a disease spreads through a population by dividing individuals into three distinct compartments: susceptible, infected, and removed. The removed compartment normally includes both deaths and recoveries; however, in this context, we assume the death rate is zero, so only recoveries are considered. The model assumes that the disease is transmitted through contact between individuals and that the population is homogeneous, meaning that everyone has the same chance of contracting the disease.

By tracking the number of individuals in each compartment over time, the SIR model enables us to make predictions about how the disease will spread and how the outbreak will evolve. To use the model, we need to know the transmission and removal rates of the disease, which can often be estimated from data or obtained from other sources. The SIR model has been widely used to study many infectious diseases, from the flu to HIV, and it continues to be an important tool for public health officials and epidemiologists.

### 2.1 SIR model with memory

In its basic formulation, the SIR model consists of three ordinary differential equations that describe how the number of susceptible, infected, and removed individuals changes over time. In our formulation, we normalize these quantities and look at how the ratios with respect to the total population change over time for each of the three compartments. The equations for the normalized quantities *s* (susceptible), *j* (infected), and *r* (removed) are given by:

(1) $$\overset{˙}{s}=-\beta sj,\overset{˙}{j}=\beta sj-\gamma j,\overset{˙}{r}=\gamma j.$$

Here the overdot indicates a time derivative and s≡s(t), j≡j(t), and r≡r(t) represent the ratio of susceptible, infected, and removed compartments, respectively, subject to the constraint that the total population remains constant, i.e., s(t)+j(t)+r(t)=1 (neglecting all birth and death rates). In this system, the parameter *β* represents the transmission rate from human to human, while *γ* is the removal rate for the disease. The basic reproduction number, defined as $R_{0}=\beta /\gamma$, is a crucial parameter to assess the transmission potential of an infectious disease. Its importance lies in its ability to determine the number of new infections generated by a single infected case, thereby establishing the likelihood of the spread of the disease within a population. To simplify the model, we introduce a time rescaling t→γt, leading to the representation:

(2) $$\overset{˙}{s}=-R_{0}sj,\overset{˙}{j}=R_{0}sj-j,\overset{˙}{r}=j$$

where the overdot now denotes differentiation with respect to the rescaled time *γt*. Such a rescaling can also improve numerical stability by avoiding issues related to very small or very large time scales, thus ensuring that the time step used in our numerical methods later on is more appropriate for the system's dynamics. This setting is commonly used in mathematical modeling as a normalization technique, allowing us to rely on fewer parameters while producing solutions for the model [35]. We also note that the proposed simplification is not uncommon within epidemiology [36], [37] as it retains the fundamental dynamics of the SIR model while making the equations more tractable. This approach focuses on the transmission dynamics represented by a single variable, $R_{0}$.

Although Equation (2) is useful in understanding the initial stages of an epidemic, it is not sufficient to predict the complex dynamics of a pandemic, such as the possibility of recurring outbreaks or multiple waves of infection due to changes in transmission rates, immune memory decay, or human behavior [15]. To overcome these constraints, [12] proposed a modification to the standard SIR model, incorporating the concept of immune memory decay. This modification accounts for the gradual loss of immunity over time and the recurrence of epidemic outbreaks. The SIR model with memory is represented by Volterra integro-differential equations (VIDEs) [38], which are capable of modeling the behavior of multiwave-type dynamics observed in recurrent outbreaks. In this framework, the term “memory” refers to the system's ability to retain information about past states, which influences current dynamics. This is achieved by introducing integral terms that account for the history of infection and removal processes. The model is then represented through

(3) $$\overset{˙}{s}=-R_{0}sj+\int_{0}^{t}K(t-\tau )j(\tau )d\tau ,\overset{˙}{j}=R_{0}sj-j,\overset{˙}{r}=j-\int_{0}^{t}K(t-\tau )j(\tau )d\tau ,$$

where *K* is a probability density function (PDF) representing the decay of the immune system. For simplicity, we denote the system in Equation (3) as follows:

(4) $$\overset{˙}{y}(t)=f(t,y(t),z(t),R_{0}),\;\;\text{where}\;\;z(t)=\int_{0}^{t}K(t-\tau )j(\tau )d\tau ,$$

where *y* denotes the vector ${\left[s,j,r\right]}^{T}$. As also stated in [12], z(t) reflects the dynamic transition from removed compartment to susceptible compartment at time *t*. We use a more general Gaussian distribution $N(\mu ,\sigma^{2})$ for *K*, with mean *μ* and variance $\sigma^{2}$ instead of the original delta distribution used in [12]. However, since the Gaussian distribution is symmetric, to ensure that K(⋅) integrates to 1 over the one-sided interval (0,T), we let $K=\frac{1}{\kappa }N(\mu ,\sigma^{2})$, where $\kappa =\int_{0}^{T}N(\mu ,\sigma^{2})d\tau$. To ensure *T* is sufficiently large to encompass all feasible values of its parameters *μ* and *σ*, we set T>t+3σ in the numerical results. This allows the memory term z(t) to be almost zero at times (e.g., when μ=t+3σ), rendering the system memoryless when appropriate.

VIDEs, such as Equation (4), offer a more detailed but flexible modeling approach compared to traditional SIRS models, which typically employ a constant rate of immunity loss, leading to regular infection waves. VIDEs incorporate memory effects, allowing the representation of gradual immunity decay over time. It has been demonstrated that such a model can simulate multi-wave forms when the kernel *K* is a delta distribution, as shown in [12]. This approach allows the model to learn the characteristics of the immunity decay process more accurately, representing different types of immunity decay function beyond the simple exponential decay assumed in many SIRS models. This capability is particularly relevant for diseases such as SARS-CoV-2, where studies have shown that antibody levels gradually decrease over several months [39]. This flexibility enables VIDEs to model various immunity decay patterns and incorporate realistic delays, such as incubation periods, resulting in a more accurate depiction of disease dynamics. Furthermore, the integrodifferential approach adapts dynamically to changes in transmission rates due to interventions or behavioral changes, making it more suitable for simulating multiwave epidemics [12].

### 2.2 The reproduction number

Forecasting epidemic dynamics requires accurately determining the critical parameter $R_{0}$. Traditionally, $R_{0}$ is considered a constant. However, this simplification often fails to capture the complexities observed in real-world data, particularly during the COVID-19 pandemic. As illustrated in Figure 4 of [15], the infection data reported reveal significant variations over time, challenging the assumption of a static $R_{0}$. To address similar issues, we extend the transmission rate *β* to be time dependent, that is, β(t), allowing us to define a time-dependent version of the basic reproduction number, as well as the effective reproduction number $R_{e}$ as follows:

(5) $$R_{0}(t)=\frac{\beta (t)}{\gamma }\;\text{and}\;R_{e}=R_{0}(t)\times s(t).$$

The effective reproduction number $R_{e}$, refers to the expected number of secondary infections caused by an infected individual at a specific time [40]. When $R_{e}$ is greater than 1, each infected individual tends to generate more than one infected individual, leading to an epidemic outbreak. The concept of dynamic transmission potential is well-supported in the literature. Diekmann et al. (2010) provide a comprehensive framework for representing transmission dynamics in complex, time-dependent settings [41]. Wallinga and Teunis (2004) introduce the effective reproduction number to reflect the impact of control measures over time, demonstrating that it can change in response to these interventions [42]. Moreover, Cori et al. (2013) offer a methodology for estimating $R_{e}$ from incidence data, highlighting its importance for real-time monitoring and control of epidemics [43]. These studies collectively show that $R_{e}$ effectively encapsulates the dynamic changes in transmission potential, which is crucial for accurate epidemic modeling and intervention planning. Following these ideas, our new, extended NODEs system becomes,

(6) $$\overset{˙}{y}(t)=f(t,y(t),z(t),R_{0}(t)),$$

where we now learn a time-dependent reproduction number $R_{0}(t)$, through a neural network (given by Equation (9)), which we describe fully in the next section. This model not only reflects the evolving nature of disease transmission but also enhances our ability to predict and respond to epidemic waves more effectively. In the next section, we present more information about producing solutions of Equation (6) estimates for the function *f* using NODEs. Inherently the methodology proposed below falls under the realm of inverse problems.

### 2.3 Parameter estimation by NODEs

Deep learning approaches have shown promise in producing numerical solutions for inverse problems. NODEs for instance is an attractive option for resolving continuous dynamics modeled by differential equations such as Equation (1). NODEs learn system dynamics using an ODE solver, such as the Runge-Kutta method. Unlike classical approaches, employing NODEs allows us to learn the unknown function described by the system by either partially or entirely replacing it by a neural network. However, to address differential equations with integral terms like z(t) in (6), we need to extend NODEs further.

The proposed model (6) contains an unknown function, $R_{0}(t)$, and three unknown constant parameters, namely *μ*, *σ*, and initial condition s(0) for susceptible individuals. Additionally, the initial condition r(0), for removed individuals, is defined as r(0)=1−s(0)−j(0) (also note that typically j(0) is known from the data). With the properties of NODEs, we can replace $R_{0}(t)$ with a feedforward neural network (FNN) and set *μ* and *σ* to be learnable parameters. The FNN is a type of artificial neural network where connections between the nodes do not form a cycle. It consists of an input layer, one or more hidden layers, and an output layer. Each layer contains neurons that process input data using weights, biases, and activation functions to produce an output. This structure allows FNNs to learn complex patterns in the data. The initial condition s(0) can be optimized using Bayesian Optimization (BO), with details to be discussed later.

Differential equations containing integrals, previously introduced as VIDEs, are much more challenging and some studies have attempted to solve them using Runge-Kutta methods [44], [45]. However, these methods can not be applied to the SIR model (6) we consider here due to, among other things, the convolution in the integral term. Specifically, the integral component *z* involves an intractable convolution term since the range of that integral extends over past, and current values of the dependent variable. This entire integral needs to be computed in advance before each Runge-Kutta step. To handle this issue, we propose an adaptation of the Runge-Kutta numerical solver for (6) as follows.

(7) $$y_{t_{n+1}}=y_{t_{n}}+h_{1}\sum_{i=1}^{m}c_{i}f(\xi_{i},y(\xi_{i}),\overset{˜}{z}(\xi_{i}),R_{0}(t)),$$

where $h_{1}$ is the time step size and $\overset{˜}{z}$ is the numerical approximation of *z*. The Runge-Kutta parameters *m*, $c_{i}$ and $\xi_{i}$ are typically chosen to achieve the least possible approximation error. In this study, we employed a second-order Runge-Kutta scheme whose details and parameter values are provided in Appendix B. We approximate the integral *z* in (6) with $\overset{˜}{z}=h_{2}\sum_{i=1}^{n}K(t_{n}-t_{i})j(t_{i})$, where $j(t_{i})$ is approximated by linear interpolation and $h_{2}$ denotes the length of the subinterval. To ensure accurate sampling of the entire curve under consideration, we set $h_{2}=\min(0.01,\sigma /3)$. This choice is based on the normal distribution's high degree of symmetry and steep drop-off on either side of the mean, suggesting that $h_{2}$ should be proportional to *σ*, as per the commonly used rule of thumb [46]. This proportionality ensures effective sampling. Clearly, smaller choices for $h_{2}$ will produce more accurate estimates at the expense of increased computational time. Our proposed methodology therefore extends the traditional NODEs approach, uniquely adapting it to handle integro-differential equations with convolutions. This novel adaptation furthermore allows for a broader application of the NODEs framework, addressing more complex equations. We use the Mean Squared Error (MSE) metric to evaluate the model's performance, defined as,

(8) $$\text{loss}=MSE(j_{data},j_{prediction})=\frac{1}{n}\sum_{i=0}^{n}{\left(j_{data}(t_{i})-j_{calibration}(t_{i})\right)}^{2},$$

where $j_{data}$ represents the ground truth data and $j_{calibration}$ represents the data generated by the NODEs within the time interval of calibration.

We approximate $R_{0}(t)$ through a feedforward neural network N(⋅), which learns the temporal changes of this crucial variable from available reported data. The parameters of N(⋅) are initialized using Kaiming initialization [47]. To improve N(⋅)'s learning capability, we input a 32-dimensional vector transformed from time *t* using the time embedding (TE) method proposed in [48], [49]. The details of the TE can also be found in Equation (12) in Appendix A. The model has 3 hidden layers, each consisting of 20 nodes with activation functions tanh. The final layer contains a single node and uses the Softsign activation function [50], x/(1+|x|), which results in constraining the output value within the range (−1,1). The Softsign activation function is chosen since it is smoother than tanh, leading to more gradual changes in the output. It is also well known that activation functions have a regularizing effect. Overall therefore $R_{0}(t)$ is learned through the neural network *N* via,

(9) $$R_{0}(t)=(N(\text{TE}(t,i))+1)\frac{\alpha }{2},$$

where *i* is the dimension index, *α* is an upper boundary of $R_{0}(t)$ and the lower boundary is 0. Furthermore, the parameters *μ* and *σ* of the Gaussian distribution K(⋅) were treated as learnable, allowing them to be optimized along with the weights of the neural network for $R_{0}(t)$.

### 2.4 Bayessian optimization

In order to estimate appropriate starting values for susceptible individuals s(0), and to achieve faster convergence of the parameters *μ* and *σ*, we employ a Bayessian Optimization (BO) technique. This method leverages a probabilistic surrogate model to efficiently balance the exploration and exploitation of expensive black-box functions, ultimately aiming for a global optimization solution. The surrogate model predicts promising areas of the hyperparameter search space and focuses evaluations in these regions up to a predefined maximum number of searches, which we define below. We have selected a Gaussian mixture model (GMM) as our probabilistic surrogate model due to its demonstrated adaptability to complex data [51].

Because we use a maximum of 347 days of data in all experiments, setting an upper bound of σ=40γ and T=480γ>(347+3⁎40)γ ensures that z(t) will be zero, i.e., memoryless, at times within this data period. To ensure rapid convergence and accurate learning of parameters, we define the search space for BO as follows: The mean *μ* ranges from $\frac{3}{\gamma }\gamma$ to *T*, meaning the average immune system decay is at least three times the duration, rescaled by *γ*. The standard deviation *σ* ranges from 0.001 to 40*γ*. The initial state s(0) ranges from 0 to 1−j(0). The basic reproduction number $R_{0}$ ranges from 0 to *α*, as previously discussed. These ranges are sufficiently large to ensure that the search space used in BO includes all feasible values for these parameters. The overall training procedure to learn μ,σ,s(0), as well as $R_{0}(t)$ from data in a given time period $\left[0,t_{end}\right]$, consists of the following steps:

1. **Initialization of**$R_{0}(t)$**:** Obtain an initial constant $R_{0}$ using BO in the 4-dimensional search space defined above that minimizes the objective function (8), with $j_{calibration}$ computed as the numerical solution of *j* in Equation (4). The BO process runs for a fixed number of 300 iterations. Once an optimal $R_{0}$ is found, we train $R_{0}(t)$ in Equation (9) to produce this $R_{0}$ over the entire period by minimizing $MSE(R_{0},R_{0}(t))$ for any $t\in [0,t_{end}]$. The Adam optimizer [52] with learning rate 1e-3 is used here.
2. **Estimate candidate***μ***,***σ***and**s(0)**using BO:** Estimate candidate values for μ,σ,s(0) using BO with a fixed number of 100 iterations on their defined search space to minimize the objective function (8). The $j_{calibration}$ is produced from the extended NODEs in Equation (6) through Equation (7) without retraining the network.
3. **Training extended NODEs in the data (without BO):** Treat *μ* and *σ* as learnable parameters. Minimize the objective function (8), with $j_{calibration}$ generated by our extended NODEs (6) as solved using Equation (7), by training the network parameters for $R_{0}(t)$ as well as *μ* and *σ* using the Adam optimizer with a learning rate of 1e-4 over 300 epochs.

**Figure 1:**
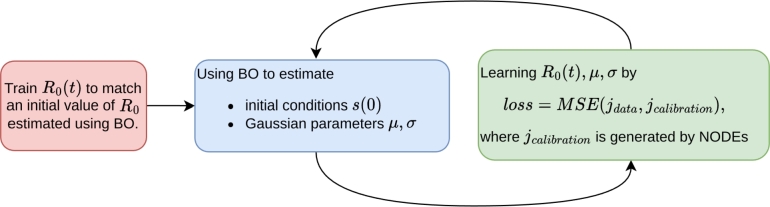
Flow chart of model training to learn *R*_0_(*t*),*μ*,*σ* and *y* = [*s*,*j*,*r*]^*T*^ using Bayesian Optimization (BO) and minimizing the loss in Equation (8) satisfying the NODEs in (6).

We repeat steps 2 and 3 above 15 times. The reason for learning *μ* and *σ* in both of these steps is that step 2 accelerates the training process and helps the model escape local minima, thus finding the appropriate values for *μ* and *σ*. We refer to Fig. 1 for a visual representation of the overall training process.

The critical difference between solving for *j* in Equation (4) or solving for *j* in the extended NODEs (6) is the treatment of $R_{0}$ as either fixed or time-varying. To compute $j_{calibration}$, we first use Bayesian Optimization (BO) to find an optimal fixed $R_{0}$ for Equation (4). This optimal value serves as a good initial condition for the neural network that represents $R_{0}(t)$ in Equation (9). We then train the neural network to match this optimal $R_{0}$ over the entire period, ensuring faster convergence. Subsequently, we solve Equation (6) using the Runge-Kutta method described in Equation (7) iteratively until convergence.

### 2.5 Evaluation metrics

To evaluate the prediction of the model, the median absolute relative percent errors (MARPE) metric was used to capture the central tendency of the distribution of errors as follows,

(10) $${\text{MARPE}}_{t}=\text{median}\left(\frac{\left|j_{data}-j_{prediction}\right|}{C}\right),$$

where $j_{prediction}$ is the predicted value within the prediction period, *t* represents a time period of exactly *n* weeks ahead of the calibration period for $j_{data}$ and $j_{prediction}$. The normalizing constant *C* is set to the maximum value over the entire data period. This metric allows us to compare prediction errors across different data scales in various countries effectively.

In Section 4, we also present the evaluation of the model's capability to predict the timing of the peak infected cases. We measure the median absolute error in days (MAED) between the predicted peak date and the observed peak date,

(11) $${\text{MAED}}_{t}=\text{median}(|{\text{Peak}}_{data}-{\text{Peak}}_{prediction}|),$$

where *t* is the time period of the model date, ${\text{Peak}}_{data}$ is the observed peak date, and ${\text{Peak}}_{prediction}$ is the predicted peak date by the model in that period. In the case that the observed peak date is constant over a period of time, a median is computed between those days.

## 3 Data

In this research, we set *γ* at a fixed value of 1/14, where the number 14 is based on multiple studies suggesting a median recovery time for COVID-19 between 8 and 20 days [53], [54], [55], [56]. This implies a time step of 1/14 in the computational model corresponds to one day in the actual data.

### 3.1 Synthetic data

To validate the efficacy of our model, we first validate it using synthetic data. We generate synthetic data using the Markovian random walker model introduced by [57] to simulate the spread of epidemics on undirected graphs. Specifically, we use a 50x50 grid graph where each node represents a location connected to its eight nearest neighbors (Moore neighborhood). This grid structure realistically simulates local interactions and the spatial spread of infection. In this model, each walker (individual) can be in one of three states: susceptible, infectious, or removed. As previously stated, we assume a median infectious state which lasts 14 days, setting the removal rate *γ* to 1/14. When an infectious walker encounters a susceptible walker at the same node, there is a probability *ρ* that the susceptible walker becomes infected. The removed walkers can become susceptible again based on a probability distribution represented by the function K(t) in the SIR model (4), reflecting immune memory. The duration of immune memory is influenced by various factors, such as the pathogen and the individual's immune system. For our simulation, we arbitrarily used a Gaussian distribution with a mean of μ=70 and a variance of $\sigma^{2}=1$, N(70,1), for K(t). The algorithm for generating synthetic data capable of producing multiwave dynamics is provided below:

1. Initialize the state of 50×50 random walkers uniformly across a 50×50 grid. Assign 1% of these walkers to the infectious state and the remaining to the susceptible state.
2. At each time step *t*, each walker can move to one of its eight nearest neighbors or remain in place. Update the state of all points. If a susceptible walker encounters an infectious walker at the same node, there is a probability ρ=2.3/14 that it will become infectious. A removed walker will become susceptible again based on a probability drawn from a N(70,1) distribution.
3. Record the proportion of susceptible, infectious, and removed walkers at each time step *t*.
4. Repeat steps 2 and 3 until t=400 days.

**Figure 2:**
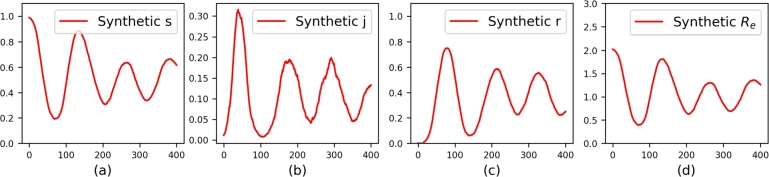
**The synthetic datasets.** (a), (b), and (c) represent synthetic *s*, *j*, and *r*, respectively. (d) shows the synthetic *R*_*e*_, defined as the synthetic *s* multiplied by *R*_0_ = 2.044.

To understand the potential spread of the epidemic in our synthetic model, we calculate the basic reproduction number $R_{0}$. This metric, in biological terms, quantifies the number of secondary infections produced by an index case in a completely susceptible population during their entire infectious period [58]. As a result, $R_{0}$ is calculated as the product of the infection probability (ρ=2.3/14), the infectious period (14 days) and $n_{e}$, the expected number of susceptible walkers that an infected walker meets per day [59]. For example, if $n_{e}=1$, an infected individual will, on average, transmit the infection to 2.3 susceptible individuals over 14 days. Given that we initialize only 1% of the population (25 out of 2500) in the infectious state, we assume that infected individuals are initially surrounded by susceptible individuals, with no other infected individuals nearby on the first day. Therefore, $n_{e}$ at the initial state of dynamics is 8/9. Consequently, $R_{0}=\frac{2.3}{14}\times 14\times \frac{8}{9}\approx 2.044$. The generated synthetic data are shown in Fig. 2. Subfigs. 2.a,b,c represent synthetic values of *s*, *j*, and *r*, respectively, while Fig. 2.d illustrates the synthetic $R_{e}$ calculated using Equation (5).

### 3.2 Real data

The COVID-19 data used for the comparisons in our study consists of two components:

• **A rolling average of 7 days of reported daily infections:** The data are based on the average daily infections found in the number of swabs taken in each of the three countries, Mexico, South Africa, and South Korea.
• **The estimated number of daily infections:** The data obtained by leveraging information from seroprevalence surveys, daily cases, and daily deaths, and, where available, daily hospitalizations during the same period for the three countries mentioned above [60].

Both datasets are freely available from the Institute of Health Metrics and Evaluation through [61] at the University of Washington.

Therefore, this paper does not aim to estimate the actual number of infections, but rather to predict the reported number of infections by learning from the published data and our proposed model in (6). However, we have tried to choose countries with high testing rates such as South Korea over a 400-day testing period starting from November 5, 2021. We note that this period ensures that the data include multiple COVID-19 infection waves. We also took data from South Africa and Mexico during the same period for comparison purposes. The starting date of 5 November 2021 is also chosen because the World Health Organization designated the new variant as a variant of concern and named it Omicron (B.1.1.529) on 26 November 2021 [62], which is first reported in South Africa. Since we assume γ=1/14, to simplify the calculation, we determine the current infectious population by adding all the cases in the past 14 days.

Naturally, all such data would be exposed to inaccuracies due to a number of issues such as changes in reporting standards for each country, incomplete or missing data, etc. Furthermore, these data would also be sensitive to the presence of asymptomatic infections [63], [64], [65], [66], [67]. In addition, in many countries where the data are sourced, the low test rate and COVID-19's incubation period of up to 27 days (see [65]) contribute to a significant undercount of daily cases compared to the actual count.

Based on published COVID-19 data, several studies [68], [69] indicate that the average basic reproduction number varies significantly between different variants of COVID-19 due to differences in their transmissibility. Specifically, the average observed values of $R_{0}$ are approximately as follows: Original Strain 2.79, Delta Variant 5.08, and Omicron Variant 9.5 as reported in [69]. To account for this variability, we set α=10 in Equation (9), which constrains the range of values for $R_{0}(t)$ to the interval [0,10].

## 4 Results and discussion

Various models incorporate a short-memory parameter to address multiple waves in observed dynamics, using approaches such as the Kalman filter (Figure 2 in [15]) or Bayesian inference [16], [25]). Other methods employ a data-driven approach for inference by allowing adaptivity in parameters through a short-term sliding window (as in [21]). Due to the short-memory structure inherent in these models, the resulting predictions are better at representing shorter day-to-day trends but often fail to capture longer-term dynamics or multiwave infections.

**Figure 3:**
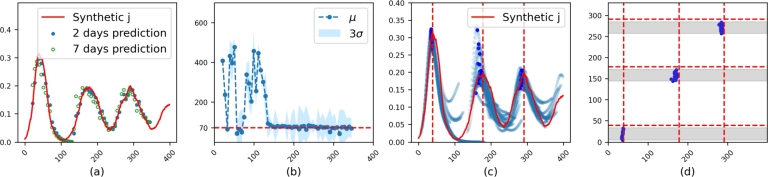
**Results on synthetic data.** The synthetic data was generated with *μ* = 70 and *σ* = 1 over 400 days. All x axes denote days. **(a)** Synthetic infection data (red) with corresponding predictions for 2 (green points) and 7 (blue points) days ahead. **(b)** Successive predictions of *μ* and *σ*. The blue points represent *μ* of each *K*(⋅), and the blue shade indicates the corresponding range of three standard deviations. The red dashed line is for actual *μ* = 70. **(c)** Successive long-term (8 weeks) prediction (blue scatter line) for *j* is provided to show the trend of dynamics. The dark blue points are predicted peaks. **(d)** The vertical red dashed lines denote the observed peaks. On the X-axis, we have predicted maximum dates, while the Y-axis represents model dates. The gray bands represent the windows of 34 to 7 days that precede each peak. For each model date within this window, we obtain a predicted peak (dark blue point).

Based on the proposed methodology described in Section 2 and the synthetic data generated in Section 3.1, we present predictions for the ratios of infected individuals, as illustrated in Fig. 3. Similar results for synthetic data with different parameter settings are provided in Appendix C. For the synthetic data, the models were trained with α=6, constraining $R_{0}(t)$ within the range (0,6). In Fig. 3.a, the synthetic infection data are depicted by the red line, along with the corresponding short-term predictions for 2 and 7 days forward. The model is trained with data from the first day up to the current day. To demonstrate the capability of the model for long-term prediction, we present the $MARPE_{t}$ (refer to Equation (10)) for predictions of 1 to 4 weeks in Table 1. As expected, longer predictions have a higher percentage of errors, but remain acceptable with less than 10%. The corresponding values of *μ* and *σ* are shown in Fig. 3.b. In particular, the memory effect indicator *μ* starts to converge to the actual μ=70 used to generate the data around the time point 120, just before the onset of the second wave. This convergence indicates persistent reinfections over time and demonstrates the model's ability to estimate *μ* using data that include a full wave and the early stages of the subsequent wave.

**Table 1:** **Weekly prediction accuracy evaluated by***MARPE*_*t*_**.** To produce the statistics presented in this table, models are trained with data chosen from the interval [0,*t*_*end*_], where *t*_*end*_ varies and is sampled to be a specific value from day 20 to day 344 with a step size of 6. This results in training 55 different models based on 55 different datasets. For each such model we generate predictions for 4 weeks ahead starting from *t*_*end*_. For predictions involving 2, 3 or 4 weeks ahead of *t*_*end*_ the mean per week of *MARPE*_*t*_ is presented. In computing (10) we let *C* be the maximum of *j*_*data*_ within the full data period [0,344].

Prediction (weeks)	Synthetic data	daily average cases	estimated cases		
		South Korea	Mexico	South Africa	South Korea
1	4.2%	2.5%	1.4%	3.5%	3.2%
2	5.5%	4.0%	2.2%	5.3%	4.8%
3	6.9%	5.9%	3.1%	7.3%	6.7%
4	7.8%	8.2%	4.2%	8.9%	8.7%

Estimating the peak infection date is crucial to understanding the progression of the pandemic and public health planning. Fig. 3.c shows long-term (8 weeks) trends in the dynamics of the underlying infection. The red dashed lines represent the actual peaks, whereas the dark blue points indicate the predicted peaks of the model. The presence of multiple blue curves, each starting on a different day, produces a profile of the infection's future trajectory, which is especially helpful for predicting the peak of the infection or the start of a new wave. As these curves overlap in their predictions, they provide more evidence for the validity of the prediction. The model captures the peaks of the first, second and third waves, as also presented in Fig. 3.d with dark blue points. For a clearer understanding, Table 2 shows the number of days between the predicted peak date and the actual peak date, calculated using Equation (11). These estimates are provided from the model daily for 7 days up to 34 days before the peak, at weekly intervals.

**Table 2:** **Evaluation of peak prediction errors using***MAED*_*t*_**.** The error is measured in terms of the number of days before or after the observed peak date and is presented at weekly intervals. To produce the statistics presented in this table, the models are trained with data within the interval [0,*t*_*end*_], where *t*_*end*_ is sampled daily from 34 days to 7 days before the peak. Week 1 corresponds to models with *t*_*end*_ between 13 days and 7 days before the peak. Week 2 corresponds to models with *t*_*end*_ between 20 days and 14 days before the peak, and so on, up to week 4. For each week, we train 7 models on a daily basis. If there is insufficient data, the results in the table are marked with an asterisk.

Model date (weeks before)		Synthetic data	daily average cases	estimated cases		
			South Korea	Mexico	South Africa	South Korea
1st peak	1	2	17	1	6	2
	2	3	19	1	10	3
	3	4	6	3	16	3
	4	5	9	18	*	7

2nd peak	1	9	19	0	3	7
	2	8	9	1	2	10
	3	9	15	2	9	8
	4	13	27	2	6	21

**Figure 4:**
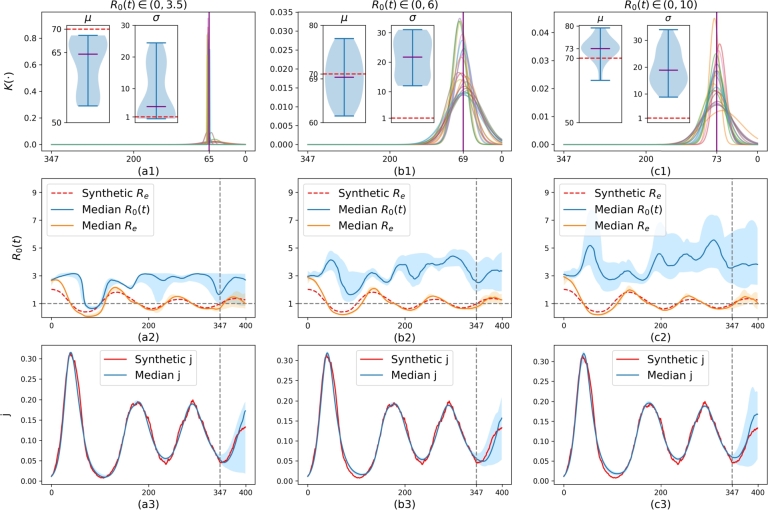
**Uncertainty of learned model on synthetic data.** The first to third columns display the results where *R*_0_(*t*) is constraint by *α* to be 3.5, 6 and 10, respectively. For each case, we train the model 20 times using the first 347 days of data. The shaded areas represent the full range covering all 20 samples. **First row:** The distribution of learned *μ*, *σ* from these 20 samples. The purple lines are median values of either *μ* or *σ*. **Second row:** The red dashed line is the synthetic *R*_*e*_, defined by synthetic *s* times 2.044. The blue line in the interval (0, 347) represents the median of the calibrated *R*_0_(*t*), and in the interval (347, 400), it represents the median of the predicted *R*_0_(*t*). The orange line is the median *R*_*e*_ that is obtained by multiplying *R*_0_(*t*) by the calibrated or predicted *s*. **Third row:** The blue line in the interval (0,347) represents the median of the calibrated *j*(*t*), and in the interval (347, 400), it represents the median of the predicted *j*(*t*).

We also notice that the constraint *α* has a significant influence on model performance and the estimation of $R_{0}(t)$. We trained the model on data from day 0 to day 347, with the period from day 348 to day 400 reserved for predictions, using three different values of *α*: 3.5, 6, and 10. Since the solution of $R_{0}(t)$ is not unique and can converge to various local minima due to the interplay between K(⋅) and $R_{0}(t)$, as well as the randomness inherent in the initial parameter settings and the Bayesian Optimization (BO) process, we generated 20 models for each case in order to illustrate the model uncertainty. The results are shown in Fig. 4. From the first row of Fig. 4 (Figs. 4.a1,b1,c1), it is clear that as *α* increases from 3.5 to 10, both *μ* and *σ* also increase. Furthermore, $R_{0}(t)$ generally has higher values as *α* increases. This makes sense since lower values of *μ* and *σ* make z(t) contribute more to generating higher values of *j*, as the memory term z(t) transfers past removed individuals back to the susceptible category. Otherwise, $R_{0}(t)$ needs to be more flexible to fit the data accurately. This can be observed in Fig. 4.c2, where the uncertainty area (blue shade) of $R_{0}(t)$ reaches as high as 7 during peak times, which is much higher than in cases with smaller *α* values as seen in Figs. 4.a2, b2. The uncertainty area (blue shade) in the second row of Fig. 4 increases as *α* increases. This indicates that when the constraint on $R_{0}(t)$ is relaxed with a higher value of *α*, we have more potential solutions, leading to increased uncertainty in the model. By examining the prediction performance between days 347 and 400 in the third row of Fig. 4 (Figs. 4.a3,b3,c3), we see that the predictions are generally better when *α* is 6 or 10 compared to when α=3.5. This suggests that a smaller *α* is not necessarily better.

All these results demonstrate that choosing an appropriate *α* is essential for the model to effectively learn from the data and make accurate predictions. The model performs very well in discovering the synthetic effective reproduction number from the data. We observe that the median of the predicted $R_{e}$ (see the orange line) is quite robust. It generally aligns with the synthetic $R_{e}$ (see the red dashed line) for different values of *α*, even when $R_{0}(t)$ varies significantly. The results are also stable, with a narrow uncertainty area (orange shade). In general, Figure 3, Figure 4 show that the proposed model can accurately identify crucial parameters *μ* and $R_{e}$ from the synthetic data.

**Figure 5:**
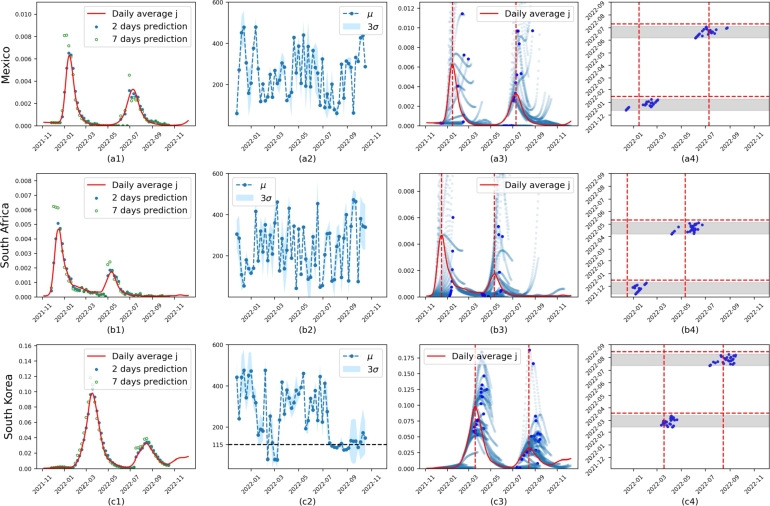
**Results on Mexico, South Africa, and South Korea datasets** based on the reported daily average cases. **First column:** presents the datasets (red line) and the predictions for 2 and 7 days. **Second column:** the corresponding successive predictions of *μ* and *σ*. The blue points represent *μ* of each *K*(⋅), and the blue shade indicates the corresponding range of three standard deviations. The convergent *μ* is represented by the horizontal black dashed line. **Third column:** Successive long-term (8 weeks) prediction (blue scatter line) for *j* is provided to show the trend of dynamics. The dark blue points are predicted peaks. **Fourth column:** the observed peaks, from row three, are displayed in vertical red dashed lines. On the X-axis, we have predicted maximum dates, while the Y-axis represents model dates. The gray bands represent the windows of 34 to 7 days that precede each peak. For each model date within this window, we obtain a predicted peak (dark blue point).

**Figure 6:**
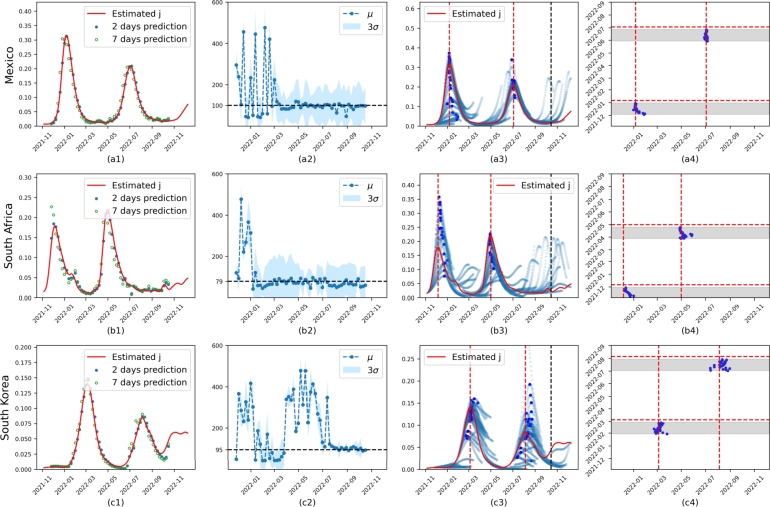
**Results on Mexico, South Africa, and South Korea datasets** based on the estimated number of cases.

To verify the performance of the model on real data, we tested it on datasets from Mexico, South Africa, and South Korea, derived from the 7-day rolling average of reported daily cases or estimated case numbers. The results are presented in Fig. 5 and Fig. 6, respectively. The first column (Fig. 5.a1,b1,c1 or Fig. 6.a1,b1,c1) presents the datasets (red line) and predictions for 2 and 7 days. The second column (Fig. 5.a2,b2,c2 or Fig. 6.a2,b2,c2) includes the successive corresponding predictions of *μ* and *σ*, with the convergence *μ* represented by the horizontal black dashed line. The successive predictions of *j* are shown in the third column (Fig. 5.a3,b3,c3 or Fig. 6.a3,b3,c3) to demonstrate the dynamic trend, including the successive predicted peak (dark blue points). The fourth column (Fig. 5.a4,b4,c4 or Fig. 6.a4,b4,c4) displays the observed peaks in vertical red dashed lines, with the predicted maximum (blue points) shown along with their corresponding predicted maximum date (X-axis) and model date (Y-axis).

The first indicator of prediction success is the parameter of the memory effect *μ*. In most cases, it converges to a number between 79 and 115, as seen in the second column of Figure 5, Figure 6. This finding is consistent with [70], which observed a rapid initial decrease in anti-N IgG levels followed by stabilization after four months. Similar results in [71] show that anti-RBD-IgG responses slowly decay over 90 days, and IgG levels remained stable for up to 3 months in samples collected up to 115 days after symptom onset (PSO) in [72].

**Figure 7:**
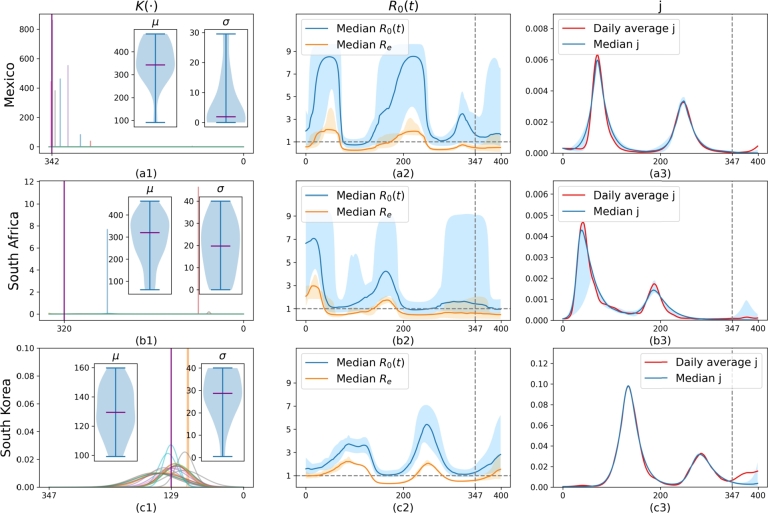
**Uncertainty of learned model on real data** based on the reported daily average cases. *R*_0_(*t*) is constraint by *α* to be 10 in all cases. For each case, we train the model 20 times using the first 347 days of data. The shaded areas represent the full range covering all 20 samples. **First column:** The distribution of learned *μ*, *σ* from these 20 samples. The purple lines are median values of either *μ* or *σ*. **Second column:** The blue line in the interval (0, 347) represents the median of the calibrated *R*_0_(*t*), and in the interval (347, 400), it represents the median of the predicted *R*_0_(*t*). The orange line is the median *R*_*e*_ that is obtained by multiplying *R*_0_(*t*) by the calibrated or predicted *s*. **Third column:** The blue line in the interval (0,347) represents the median of the calibrated *j*(*t*), and in the interval (347, 400), it represents the median of the predicted *j*(*t*).

In instances where *μ* converges, as in Fig. 3.b, Fig. 5.c2 and all cases in Fig. 6, then the method succeeded in finding the model parameters and can produce useful predictions. However, in the cases of Mexico and South Africa, where *μ* did not converge (see Figs. 5.a2, b2), the model seems to have difficulty capturing sufficient information from the reported infection history to correctly infer the second wave. This issue arises because the scale of *j* is too small for the loss function to recognize the importance of the memory term in second waves. However, in these cases, $R_{0}(t)$ plays a more significant role in the fitting of the data, as illustrated in Figs. 7.a2 and b2, which show highly variable $R_{0}(t)$ values. This inability for *μ* to converge may be attributed to external factors not included in the model. For example, Mexico changed its data collection policy in January 2022, affecting the reported average daily cases.

To better understand prediction errors, we compute metrics such as the median absolute relative percent errors (MARPE) in Table 1 and the median absolute percent errors (MAPE) in Table 3 in the Appendix. We only compute these estimates for the cases where the model parameters were possible to identify from the training data - as indicated by convergence to the mean *μ*. These tables serve as tools for assessing the precision of our weekly predictions against actual data. Our findings align closely with those of synthetic data: longer-term predictions exhibit higher errors but generally remain below 10% as can be seen in Table 1.

Fitting the dynamics of multiwave infections, such as COVID-19, presents a complex challenge. The generalized SIR model proposed here reproduces wavelike dynamics similar to those observed in pandemic data (Figure 3, Figure 5, Figure 6). Accurate predictions require sufficiently long infection dynamics for better learning of z(⋅). Initially, the model encounters difficulties near the end of the first wave but correctly follows the trend once *μ* starts to converge (the first two columns in Figure 5, Figure 6). For convergent cases, we estimate the dates of each wave peak in Table 2. The error generally converges (smaller) from week 4 to week 1, with higher errors in South Korea. Similar results are found in Figure 7 of [73], which used mortality data for the predictions. Figs. 5.a3 and b3 exhibit exponential growth, overshooting the peak around January 2022. Similar exponential growth predictions are found in [73] (Fig. 6) and [74]. The authors in [74] explain that such predictions “can occur if the mutation rate of a virus is proportional to the current number of infections,” incorrectly predicting a “giant infection wave.”

With large enough infections, long-term inferences are possible. Based on a sufficiently long period of ascent, the model produces reasonable long-term estimates of the temporal occurrence, amplitude, and decay of the first wave. Similarly, if training datasets include the descent of the first wave and the beginning of the second wave, the model can predict the ascent toward the second peak, as seen in the third column of Figure 5, Figure 6. As long as *μ* is convergent, the model can naturally infer even a third infection wave, as seen in Figs. 6.d3, 6.e3 and 6.f3. Discrepancies in the prediction of the third wave, seen in Fig. 6.e3, may depend on known reporting restrictions in our input data, as discussed below.

**Figure 8:**
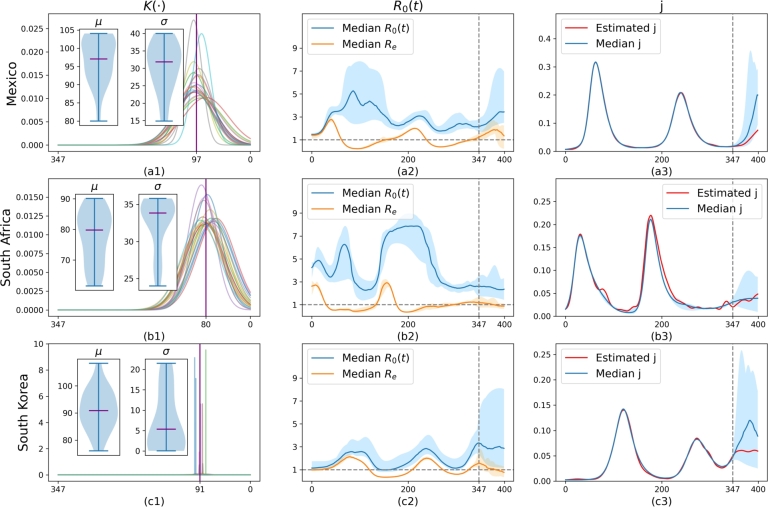
**Uncertainty of learned model on real data** based on the estimated number of cases.

To demonstrate the capability of the model to fit $R_{0}(t)$ or $R_{e}$, we trained the model 20 times for each case using data from the period 0 to 347. The results based on the average daily reported data and estimated case numbers are shown in 7 and 8, respectively. The first column (Figs. 7.a1,b1,c1 or Figs. 8.a1,b1,c1) shows the learned distributions. The second column (Figs. 7.a2,b2,c2 or Figs. 8.a2,b2,c2) displays the uncertainty of calibrated or predicted $R_{e}$ and $R_{0}(t)$. The third column (Figs. 7.a3,b3,c3 or Figs. 8.a3,b3,c3) presents the uncertainty of calibrated or predicted j(t). The model performs well in fitting the data even for cases where *μ* of z(⋅) does not converge (Fig. 7.a3,b3). This success is attributed to the fact that $R_{0}(t)$ learns to exhibit two waves, as can be seen in Figs. 7.a2 and b2, thus compensating for potentially incorrect z(⋅) values. In contrast, other regions do not show distinct high uncertainty, as indicated by the smaller blue-shaded areas (Fig. 7.c2, Fig. 8.a2, b2, c2). For these cases, the median value of the learned $R_{0}(t)$ generally remains below 6, except in Fig. 8.b2, where it increases during the second wave. Despite the large uncertainty in $R_{0}(t)$ (blue-shaded area), $R_{e}$ is much more stable, with a relatively small uncertainty area (orange-shaded area) that is sometimes barely visible. This stability holds even for the first two cases. We can see that infections increase rapidly when $R_{e}>1$. These results demonstrate that $R_{e}$ is a more stable and potentially more useful indicator for such a model (6) for pandemic prediction.

Policy changes at the national level may have contributed to decreased COVID-19 testing rates, resulting in less reliable input data. For example, many regions revised their COVID-19 restriction policies at the end of 2021. Mexico removed entry requirements in January 2022, South Africa on June 23, 2022, and South Korea in October 2022. Furthermore, “since December 2021, the Omicron variant and its subvariants have been circulating in Mexico” [75], and “by June 2021, the pandemic was dominated by the Delta variant” [75]. The emergence of these new variants and the opening of borders undoubtedly changed the status of the input data.

In general, a significant challenge in our estimation and prediction models was the incomplete nature of the available data. Specifically, our models are based on reported symptomatic infections that can be subject to delays or occur within censored time intervals. This incomplete data can hinder the accuracy of our predictions and the reliability of our estimates. Additionally, the model is designed to predict multi-wave pandemics, and it may not be helpful if the data does not contain peaks. However, the method of estimating $R_{0}(t)$ can still be applied to other types of SIR models. Future work will aim to address these limitations by incorporating methods to account for underreporting and reporting delays, such as data imputation techniques or integrating supplementary data sources, to enhance the robustness and precision of our models.

## 5 Conclusions

Although World Health Organization (WHO) experts have declared that COVID-19 is no longer a global health emergency [76], COVID-19 continues to spread and the virus remains a global health threat as it evolves.

In this work, we proposed a solution methodology to resolve a generalized SIR model with memory in order to reproduce some of the characteristics of the infection dynamics found in the data. Our proposed hybrid approach combines machine learning as well as classic numerical methods, effectively solving an inverse problem to generate critical model parameters as well as the system solution through an improved optimization process. Specifically, we proposed a strategy based on Bayessian optimization together with a Runge-Kutta classical differential equation solver that resolves a generalized version of NODEs [13] to produce adaptive, time dependent, parameters for a modified SIR model with memory.

The generalized SIR model proposed here includes a long-memory parameter z(⋅), which represents the decay of the immune system, as well as a time-varying effective reproduction number, $R_{e}$. For our methodology, both quantities are estimated from the data through the proposed network. The effectiveness of the model is first evaluated by testing it on synthetic data. Then it was further tested on officially estimated as well as officially reported data from Mexico, South Africa, and South Korea. Solutions produced from this model based on the data from these countries allow us to forecast multi-wave infection dynamics as well as some of the SIR model critical parameters. Parameters such as the memory effect indicator *μ* as well as a continuous, and many times non-smooth, effective reproduction number $R_{e}$ can be crucial in understanding infection trends. In all cases for which the parameters converged, the model was able to reproduce the expected multiwave infection dynamics of COVID-19. This also allows us to produce estimates of the next peak of the infection.

One of the limitations of the proposed approach is that when the amount of infected data is much smaller than the population, then *s* and *r* become almost constant. This makes it very difficult to learn important parameters such as *μ* and the memory factor z(⋅) because they may not converge. This was the case, for example, for the reported infection data obtained from Mexico and South Africa. More data pre-processing may be needed as an option to counteract this lack of data. Alternatively, to somewhat counteract this data discrepancy, methods such as normalizing flows [77] could also be considered to improve estimates of the unknown distribution space where the infection data came from. Another idea that could further improve the predictions would be to also allow the memory parameter *μ*, which for now is assumed to be a constant, to be a vector. In effect, that would allow us therefore to learn several distributions within the data through a mixture Gaussian method. Such adaptations could allow sufficient freedom in that parameter space in order to capture more realistic distributions and dimensionality in the data.

As also suggested in [17], we hope that the proposed model and approach can be useful as part of an ensemble of models to be used to establish possible future directions of infectious dynamics. For future work, we also propose integrating vaccination effects in the model dynamics by incorporating compartments for vaccinated individuals and considering reduced transmission rates among the vaccinated population. The model can be adjusted using methods similar to those discussed in [78], where vaccination parameters are included to reflect the impact on disease dynamics.

In future work, our aim is to further explore combinations of classical and data-driven modeling approaches to enhance our understanding and prediction of infectious disease dynamics. One such approach could combine, for instance, Markov Chain Monte Carlo (MCMC) methods alongside the data-driven techniques such as the one proposed here. We will then integrate these models with data-driven approaches, which excel in handling the complex and less understood parts of the dynamics that are nevertheless captured in available data. Such hybrid models can leverage the strengths of both methodologies: the robustness and theoretical foundation of classical models, and the flexibility and predictive power of data-driven techniques. We believe that this integrative approach could broaden the scope of our modeling capabilities and enhance the overall accuracy and reliability of our predictions. By systematically combining well-established methods with innovative data-driven strategies, our goal is to advance the science of epidemiological modeling, ultimately contributing to more effective public health interventions and policies.

## CRediT authorship contribution statement

**Donglin Liu:** Writing – review & editing, Writing – original draft, Visualization, Validation, Methodology, Investigation, Formal analysis. **Alexandros Sopasakis:** Writing – review & editing, Writing – original draft, Project administration, Methodology, Investigation, Funding acquisition, Formal analysis, Conceptualization.

## Declaration of Competing Interest

The authors declare the following financial interests/personal relationships which may be considered as potential competing interests: Donglin Liu reports financial support was provided by eSSENCE. Donglin Liu reports financial support was provided by Swedish Research Council Formas. Alexandros Sopasakis reports financial support was provided by Swedish National Space Board. Alexandros Sopasakis reports equipment, drugs, or supplies was provided by Lunarc. If there are other authors, they declare that they have no known competing financial interests or personal relationships that could have appeared to influence the work reported in this paper.

## Figures and Tables

**Figure 9:**
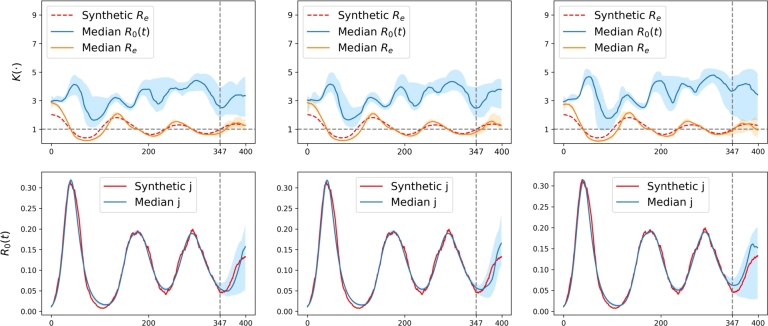
**Uncertainty of the learned model on synthetic data across different neural network architectures.** Each column represents results from a different neural network architecture. The first column shows the results for a model with 3 hidden layers, each consisting of 20 nodes. The second column presents the results for a model with 3 hidden layers, each consisting of 32 nodes. The third column displays the results for a model with 5 hidden layers, each consisting of 20 nodes. In all cases, *R*_0_(*t*) is constrained by *α* = 6 and the model is trained 20 times using the first 347 days of data. The shaded areas represent the full range covering all 20 samples.

**Table 3:** **Weekly prediction accuracy evaluated by***MAPE*_*t*_**.** To produce the statistics presented in this table, models are trained with data chosen from the interval [0,*t*_*end*_], where *t*_*end*_ varies and is sampled to be a specific value from day 20 to day 344 with a step size of 6. This results in training 55 different models based on 55 different datasets. For each such model we generate predictions for 4 weeks ahead starting from *t*_*end*_. For predictions involving 1, 2, 3 or 4 weeks ahead of *t*_*end*_ the mean per week of *MAPE*_*t*_ (refer to Equation (13)) is presented.

Prediction (weeks)	Synthetic data	daily average cases	estimated cases		
		South Korea	Mexico	South Africa	South Korea
1	19.7%	22.8%	12.8%	18.9%	19.2%
2	24.3%	30.7%	17.7%	27.1%	25.2%
3	29.61%	39.6%	23.1%	37.2%	31.3%
4	34.1%	49.6%	29.2%	48.6%	37.5%

**Figure 10:**
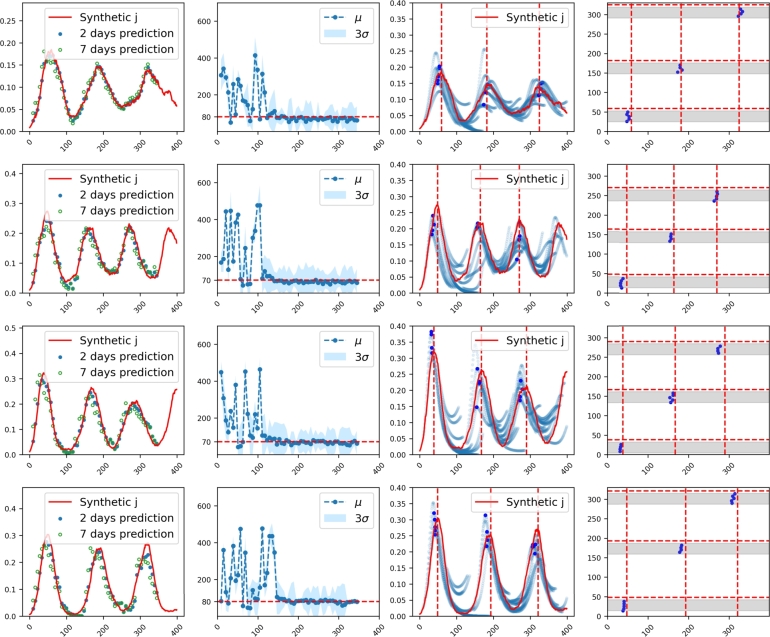
**Results on multiple synthetic datasets with different parameter settings.** First row: results on synthetic data with $\rho =\frac{2.0}{14}$ and *μ* = 80. Second row: results on synthetic data with $\rho =\frac{2.5}{14}$ and *μ* = 70. Third row: results on synthetic data with $\rho =\frac{2.7}{14}$ and *μ* = 70. Fourth row: results on synthetic data with $\rho =\frac{2.9}{14}$ and *μ* = 80.

## Data Availability

The data that support the findings of this study are openly available at https://covid19.healthdata.org/. The code is available at https://github.com/lindliu/IDE.git.

## Appendix A

*Time embedding*

Time embeddings are a way to encode the position of data points (in this case, time points) into a higher dimensional space, allowing a model to capture temporal dependencies and relationships. The use of sine and cosine functions for this purpose is inspired by the positional encoding used in transformer models, as described in works such as Vaswani et al. (2017) and further elaborated in other research such as Zuo et al. (2020), [48], [49]. Practically, however, the smooth, continuous nature of sine and cosine functions helps in maintaining the continuity of the temporal information, making it easier for the model to learn patterns over time.

Specifically we employ the following time embedding (TE) using sine and cosine functions of different frequencies:

(12) $$TE(t,i)=\left\{ \begin{array}{cc} \sin\left(\frac{t}{10000^{2i/d_{model}}}\right) & \text{if }i\text{ is even},\\ \cos\left(\frac{t}{10000^{2i/d_{model}}}\right) & \text{if }i\text{ is odd} \end{array}\right.$$

where *t* denotes the time or position, *i* is the dimension index, and $d_{model}$ is the total dimension of the model. We choose $d_{model}$ to be 32 in this example. We also note that the practical consequence of using different frequencies is to allow the model to capture both short-term and long-term dependencies in the data.

*Sensitivity of network architecture and optimizer*

The neural network consists of 3 fully connected hidden layers, each containing 20 neurons. The activation function used after each layer is the hyperbolic tangent (tanh), chosen for its smoothness and non-linearity, enabling the model to capture complex temporal patterns. The input to the network is a 32-dimensional time-embedded vector, which encodes time information using sine and cosine functions as described in Equation (12) in the subsection above. The final output layer consists of a single node with the Softsign activation function, which constrains the output within the range (-1, 1), while also facilitating smooth transitions in the $R_{0}(t)$ estimates.

The neural network is initialized using Kaiming initialization [47], designed for networks with nonlinear activations, such as tanh, to ensure an optimal starting point for the learning process. For training, the Adam optimizer is employed with default settings of $\beta_{1}=0.9$ and $\beta_{2}=0.999$. A learning rate of $1\times 10^{-3}$ is used during the initial optimization steps, with a reduction to $1\times 10^{-4}$ during the fine-tuning phase, as described in Section 2.4.

The sensitivity of the model to the number of neurons, the depth of the network, and the learning rate was also evaluated on the synthetic data set. We modified the neural network architectures by either increasing the number of neurons per layer from 20 to 32 or adding additional hidden layers from 3 to 5. The results did not show significant differences and overall all cases produced similar medians as shown in Fig. 9. The case of 3 hidden layers, each consisting of 32 nodes (the second column in Fig. 9), displayed slightly less variation than the other cases. Adjusting the learning rate within the range of $\left[10^{-3},10^{-5}\right]$ resulted in a variation in convergence speed, with a rate of $10^{-3}$ yielding the best trade-off between speed and accuracy.

The final model provides reliable predictions of $R_{e}$, ensuring accurate parameter estimates for the time-dependent dynamics of the SIR model. This approach, combined with Bayesian optimization for the selection of key parameters such as *μ* and *σ*, enables robust and generalizable solutions across different datasets.

*MAPE*

The median absolute percent errors (MAPE) [73], provided below, are another common metric used to explore differences between predicted and actual infections,

(13) $${\text{MAPE}}_{t}=\text{median}\left(\frac{\left|j_{data}-j_{prediction}\right|}{j_{data}}\right).$$

As can be seen in Table 3 (results based on Equation (13)), the least errors occur for predictions from synthetic data, as well as for the data based on estimated cases reported from Mexico and South Korea. We have higher errors in our predictions from estimated data in South Africa and even higher errors for data based on the daily average cases in South Korea. We refer to Section 4 for some of our consideration regarding these results. For comparison, weekly prediction results with similarly high (or even higher) errors have also been reported in Supplementary Fig. 3 on mortality data from COVID-19 in [73]. Although COVID-19 mortality data are almost exact and were reported with much higher precision by authorities during the pandemic, the resulting prediction errors in that Supplementary Fig. 3 range from 25% to, even, 100% in week 1, depending on the location, and increase from there as they try to produce predictions of mortality for subsequent weeks.

## Appendix B

*The second order Runge Kutta method*

The second-order Runge-Kutta method used in this manuscript is equivalent to the explicit midpoint method, also known as the modified Euler method [79]. The explicit midpoint method is superior to Euler's method because it utilizes a slope estimate at the midpoint of the prediction interval. The formulas are shown as follows:

$$y_{n+1}=y_{n}+hk_{2}\;\;\text{where,}\;k_{1}=f(t_{n},y_{n})k_{2}=f(t_{n}+\frac{h}{2},y_{n}+\frac{h}{2}k_{1}).$$

Here, *h* is the step size, $t_{n}$ denotes the current step, $y_{n}$ is the current value, $y_{n+1}$ is the predicted value. To numerically solve Equation (4) or Equation (6) using this numerical scheme, we choose h=1/14. This step size is sufficiently small to produce a stable solution within the time interval t∈[0,400/14] in the simulations presented in this work.

Equation (7) is employed to numerically approximate the solution of Equation (6). This involves a second-order Runge-Kutta scheme, where the integral term z(t) is approximated using a discrete sum. The step size *h* is chosen to ensure stability and accuracy. The neural network representing $R_{0}(t)$ is initialized using the optimal $R_{0}$ obtained from Bayesian Optimization, and then trained iteratively along with the Runge-Kutta updates to capture the time-varying dynamics of the model.

## Appendix C

To further demonstrate the robustness and generalizability of our method, we present additional results in Fig. 10 for various other values of *ρ* and *μ*. These results explore the influence of varying the parameter *ρ* on the model's performance, expanding beyond the initial value of $\frac{2.3}{14}$. This analysis confirms that the method consistently estimates the parameter *μ* accurately and reliably predicts subsequent peaks across different values of *ρ*, reinforcing the findings presented earlier.
